# Isolated Rem Sleep Behavior Disorder: A Model to Assess the Overnight Habituation of Emotional Reactivity

**DOI:** 10.3390/clockssleep7010009

**Published:** 2025-02-28

**Authors:** Caterina Leitner, Viviana Greco, Francesca Casoni, Penelope A. Lewis, Luigi Ferini-Strambi, Andrea Galbiati

**Affiliations:** 1 Faculty of Pyschology, “Vita-Salute” San Raffaele University, 20127 Milan, Italy; caterina.leitner@hotmail.com (C.L.);; 2 Department of Clinical Neurosciences, Neurology—Sleep Disorders Center, IRCCS San Raffaele Scientific Institute, 20127 Milan, Italy; casoni.francesca@hsr.it; 3 Cardiff University Brain Research Imaging Centre (CUBRIC), School of Psychology, Cardiff University, Cardiff CF24 4HQ, UK; vivianagreco22@gmail.com (V.G.);

**Keywords:** sleep, emotion regulation, REM behavior disorder, emotional memory, REM sleep, REM sleep fragmentation, sleep spindles, International Affective Picture System

## Abstract

(1) Background: Phasic events in rapid eye movement (REM) sleep are a core feature of isolated REM behavior disorder (iRBD), which is often associated with emotion dysregulation. This study explores the relationship between sleep and the overnight habituation of emotional reactivity in healthy controls (HCs) and iRBD patients, focusing on the role of REM phasic events and a specific non-REM waveform, namely sleep spindles. (2) Methods: Participants underwent polysomnography and completed arousal rating tasks and mood scales before and after sleep. In total, eight HCs (4 M, mean age 60.62 ± 6.8) and eight iRBD patients (7 M, mean age 68.25 ± 5.12) were included in the analyses. (3) Results: In HCs, longer REM sleep duration correlated positively with overnight habituation. In the whole sample, overnight habituation negatively correlated with REM sleep latency and wake-after-sleep onset, and positively with N2 sleep. Higher overnight habituation was associated with fewer REM arousals and awakenings in the whole sample, and with greater N2 sleep spindle density in HCs. (4) Conclusions: Our preliminary results suggest that REM sleep and spindles in N2 play critical roles in emotional processing. The study confirms the relationship between emotion dysregulation and REM phasic events, enhancing our understanding of how sleep impacts emotional reactivity and also in the prodromal phase of neurodegenerative disease.

## 1. Introduction

Sleep is a complex and active state characterized by different oscillatory activities, based on which it is possible to distinguish distinct sleep phases: Rapid eye movement (REM) and non-REM (NREM) sleep. This latter is further subdivided into three stages: from the lighter stage 1 to the deeper stage 3. REM sleep, for its oscillatory pattern, is similar to wakefulness and it is characterized by muscle atonia and rapid eye movements, from which it takes its name. There is a clear intimate connection between sleep and the emotional brain, but the role of specific sleep stages in emotional processes is less understood [1]. Neuroimaging studies have shown increased activity during REM sleep in brain regions associated with diurnal emotional experiences, such as the amygdala, hippocampus, and anterior cingulate cortex [2,3,4,5]. The reactivation during sleep of brain activity patterns elicited while learning, a natural phenomenon known as memory reactivation, forms the basis of memory consolidation [6,7,8]. In the case of emotional memories, a dual process has been hypothesized: the memory content is strengthened, while simultaneously, the emotional tone is attenuated [9]. According to the Sleep to Forget Sleep to Remember (SFSR) hypothesis by Walker, REM sleep specifically supports this decoupling due to its unique neurobiological characteristics [10]. However, other studies suggest an opposite effect of REM sleep, indicating that REM sleep duration may enhance emotional reactivity [11,12]. This view aligns more closely with the Emotional Salience Consolidation (ESC) account, which posits that REM sleep strengthens the emotional salience of memories, at least initially [11,12]. The conflicting results about the role of REM sleep in modulating emotional reactivity may be better understood considering that the decoupling of affective tone from memory content is a long process requiring several nights to be completed [13,14]. Moreover, the complexity of this process seems to require an interaction between stages during multiple NREM-REM cycles [1,15]. In particular, sleep spindles (SSs), bursts of neural oscillatory activity with a frequency between 12 and 15 Hz characterizing NREM sleep, may support the initiation of the memory reactivation phenomenon due to their frequency that facilitates plasticity processes, then the transformation of the memory may be driven by REM sleep [9,16,17].

Notably, alterations in REM sleep have been observed in several mood disorders, further highlighting the importance of REM sleep in emotional processes [18,19]. Additional evidence of the critical role of REM sleep comes from studies on sleep loss and sleep restriction, which have demonstrated that these conditions can lead to alterations in multiple domains of emotions the following day [1,20]. Moreover, numerous sleep disorders co-occur with mood symptoms. Among these, insomnia disorder, the most prevalent sleep disorder [21], often co-occur with depression [22]. Sleep fragmentation, a fundamental characteristic of insomnia, might explain this relationship [16,23]. Wassing and collaborators [16] explored the relationship between REM sleep and emotional modulation processes by studying a general population sample with varying insomnia symptoms. They found that the overnight decrease in amygdala reactivity was proportional to the total duration of REM episodes. However, this effect was nullified by maximal REM interruptions, indicating that restless REM sleep—characterized by a high number of phasic events—interferes with the overnight resolution of emotional distress. In fact, during NREM sleep noradrenaline (NA) levels are high, supporting long-term potentiation processes. Then, crucial neurobiological changes occur during REM sleep, causing the inhibition of *locus coeruleus* and subsequently a decrease in NA levels. Simultaneously, the cholinergic system becomes more active. These changes support depotentiation processes, allowing a bidirectional plasticity state. This process, together with the increasing activity during REM of limbic and paralimbic structures, seems to facilitate amygdala adaptation and its role in emotion regulation. When REM sleep is disturbed by phasic events, bidirectional plasticity is hindered and it may affect amygdala adaptation processes, enhancing the risk of emotion dysregulation.

Another sleep disorder characterized by REM sleep alterations is REM sleep behavior disorder (RBD). RBD is a parasomnia marked by vigorous dream enactment behaviors due to the loss of physiological muscle atonia during REM sleep [24]. The presence of REM sleep without atonia (RSWA) is a core feature of the diagnosis and comprehends numerous phasic events. Phasic events interrupt REM sleep, resulting in a condition named “restless REM sleep”, characterized by a high number of phasic events hindering overnight adaptation of limbic circuit activity and likely leading to emotion-related daytime impairments [25,26]. Therefore, the RBD clinical population, which is naturally rich in phasic events during REM sleep, could be a good model to test the relationship between phasic events and emotion habituation processes. Moreover, RBD in its isolated form (iRBD, i.e., when not caused by other medical or neurological conditions [27]) has significant prodromal implications, with over 90% of individuals at risk of developing synucleinopathies, such as Parkinson’s Disease (PD) and Dementia with Lewy Bodies (DLB), in 15 years [28]. Importantly, mood symptoms often precede the onset of PD and DLB [29]. Furthermore, psychiatric comorbidities, particularly depression and anxiety, significantly contribute to disability and reduced quality of life in synucleinopathies, highlighting the need for early recognition and treatment [30,31]. As a prodromal phase, iRBD offers a unique window for investigating the mechanisms underlying mood symptoms long before the overt conversion to a neurodegenerative disorder. Additionally, iRBD serves as a model for studying the relationship between phasic events during REM sleep and overnight emotional habituation processes in the context of aging and neurodegeneration. This study aims to assess the overnight habituation of emotional reactivity in healthy controls (HCs) and iRBD patients. The specific aims of this study are as follows:

- to explore the relationship between distinct sleep stages, with a particular focus on REM sleep and SSs, and the overnight habituation of emotional reactivity in a sample of elderly individuals, including both HCs and iRBD patients;
- to evaluate the relationship between phasic events in REM sleep and alterations in overnight habituation of emotional reactivity;
- to investigate emotional dysregulation in iRBD patients using questionnaires and an arousal rating task.

This study seeks to shed light on the fascinating process of overnight habituation of emotional reactivity and its alterations, providing new insights into the emotional sleeping brain in the context of ongoing neurodegenerative processes.

## 2. Results

After excluding one participant due to a headphone malfunctioning, we analyzed the data of eight HC volunteers (four male subjects, mean age 60.62 ± 6.8 years) and eight patients with iRBD (seven male patients, mean age 68.25 ± 5.12 years). Of note, we excluded the task data from one iRBD patient who did not understand the instructions, while retaining the remaining other data information. The two groups did not significantly differ in terms of gender (*p* = 0.133) and age (*p* = 0.051), although the latter is very close to the significance level. Polysomnographic (PSG) sleep parameters for each group, both for HCs and patients, are reported in Table 1.

The comparison of PSG sleep parameters between the two groups revealed significant differences: for macrostructure, REM sleep latency was longer in iRBD patients compared to HCs, as well as the time spent to reach the first epoch of NREM sleep stage 1 (N1), with the difference in sleep onset latency (SOL) to NREM sleep stage 2 (N2) being near-significant; for microstructure, the density of REMs and the number of arousals in REM sleep were higher in iRBD patients than in HCs. Given the non-significant but relevant difference between the two groups in terms of age, we performed a correlation analysis between sleep variables and age to assess whether age may influence sleep parameters. REMs density significantly and positively correlated with age (r_s_ = 0.66, *p* = 0.007). Therefore, we compared the two groups using the residuals corrected for age, performing a Mann–Whitney U test. The difference between the two groups remained significant after adjusting for age (*p* = 0.026).

Table 2 displays the comparison of the mood questionnaire scores between the two groups. No mood questionnaires were found to be significantly different between groups.

No overnight habituation effect was found in the whole sample (Wilcoxon signed-rank test statistic (W) = 25, *p* = 0.090), nor did we find any evidence that gender influenced this variable (Mann–Whitney U statistic (U) = 22, *p* = 0.768). In the comparison between iRBD and HC groups, we did not observe a significant difference in the overnight habituation index assessed with the task (U = 31, *p* = 0.779). Additionally, within each group, we did not find a significant difference in arousal scores to negative images between pre- and post-sleep (HC group: W = 9, *p* = 0.250; iRBD group: W = 4, *p* = 0.208).

**Figure 1:**
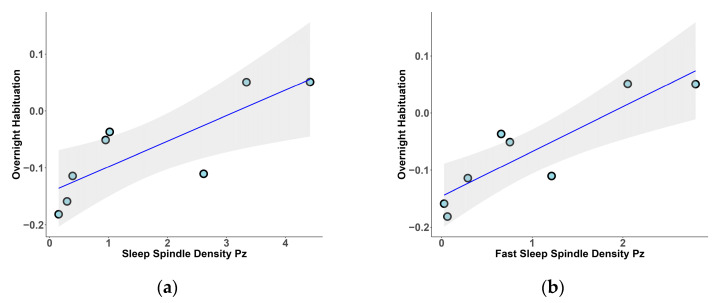
Relationship between sleep spindle density and overnight habituation in HC group: (**a**) sleep spindle density in Pz.; (**b**) fast sleep spindle density in Pz.

After evaluating the differences between the two groups, we explored the association between the overnight habituation index and the PSG sleep parameters. We observed a positive correlation between overnight habituation and N2% (r_s_ = 0.53, *p* = 0.044), as well as a negative correlation between overnight habituation and wake-after-sleep onset (WASO) (r_s_ = −0.61, *p* = 0.017). Within the HC group, we found positive significant correlations between overnight habituation and SS indices, specifically, between overnight habituation and SS density in Pz (r_s_ = 0.93, *p* = 0.02; Figure 1a). Then, distinguishing between fast and slow SSs, we found that overnight habituation positively correlated with fast SS density in Cz (r_s_ = 0.81, *p* = 0.022) and Pz (r_s_ = 0.86, *p* = 0.011; Figure 1b), and with slow SS density in Pz (r_s_ = 0.76, *p* = 0.037).

**Figure 2:**
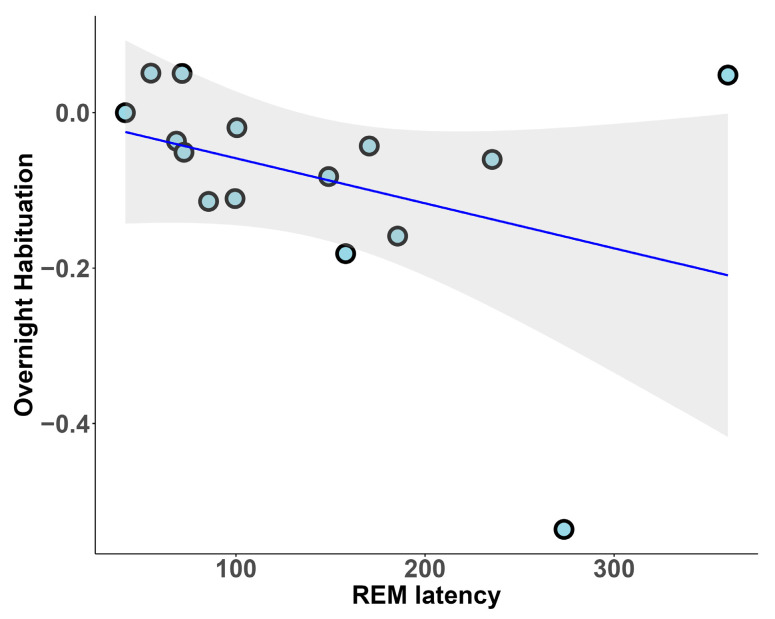
REM sleep latency and overnight habituation.

The analysis of REM macrostructure revealed that overnight habituation was significantly associated with REM sleep latency (r_s_ = −0.486, *p* = 0.034; Figure 2) but not with REM sleep duration in the whole sample (*p* = 0.593).

**Figure 3:**
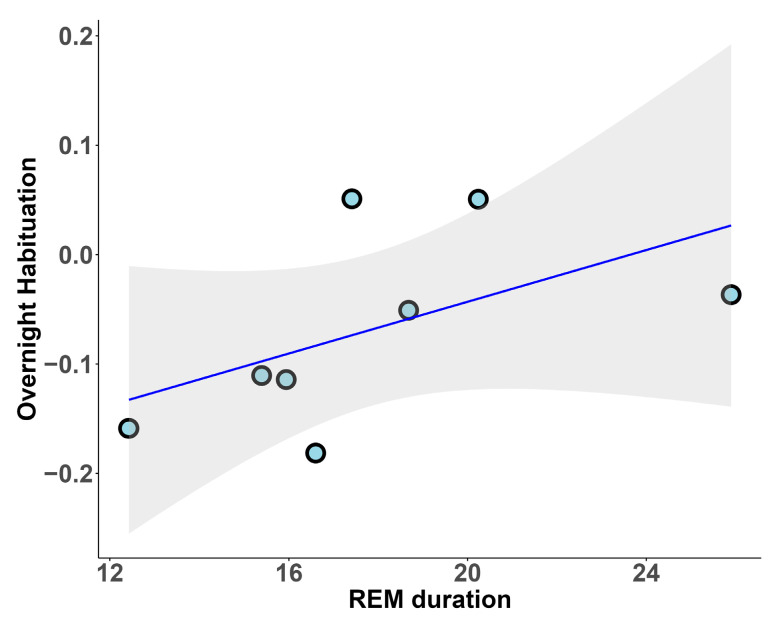
REM sleep duration and overnight habituation in the HC group.

Notably, when considering only the HC group, we found a positive correlation between REM sleep duration and overnight habituation (r_s_ = 0.67, *p* = 0.042; Figure 3).

**Figure 4:**
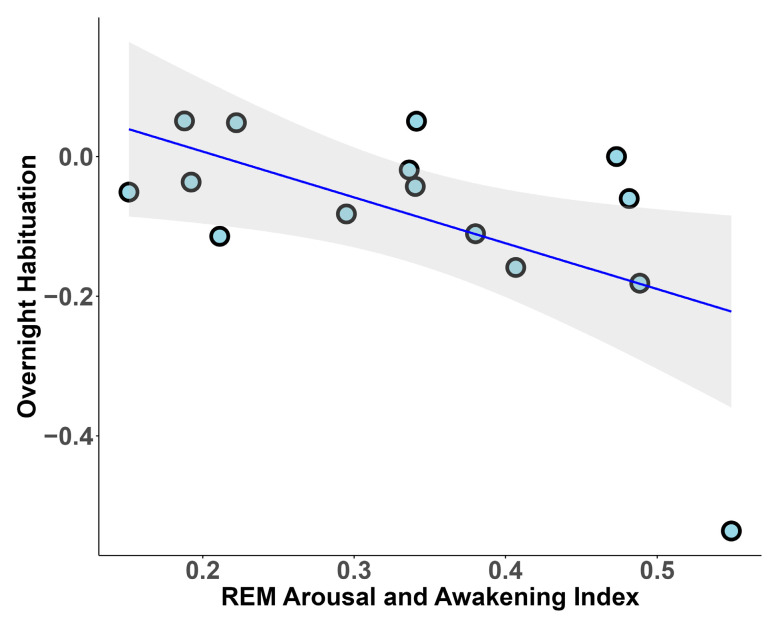
Association between overnight habituation and REM microstructure.

As regards REM microstructure, we found a negative correlation between overnight habituation and REM arousal and awakenings index (r_s_ = −0.496, *p* = 0.031) (Figure 4). Moreover, we also found a negative correlation between overnight habituation and arousal index close to significance (r_s_ = −0.43, *p* = 0.056).

**Figure 5:**
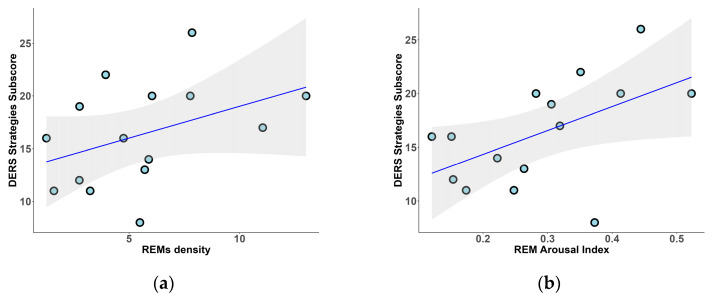
Relationship between DERS strategies subscale and REM sleep microstructure: (**a**) REMs density index; (**b**) REM arousal index.

The relationship between emotion dysregulation and phasic events was also confirmed using questionnaires to assess emotional dysregulation. Specifically, we found significant positive correlations between the strategies subscale score of the difficulties in emotion regulation scale (DERS) and REM phasic indices, including REMs density (r_s_ = 0.51, *p* = 0.049; Figure 5a) and REM arousal index (r_s_ = 0.55, *p* = 0.035; Figure 5b).

## 3. Discussion

The present study aimed to investigate the relationship between sleep and the overnight habituation of emotional reactivity in a sample of elderly individuals, including both HCs and iRBD patients, with a particular focus on the interplay between REM and NREM stages. We hypothesized a beneficial role of SSs in NREM and a detrimental role of REM phasic events on overnight habituation processes.

In summary, the results revealed significant differences between the two groups in relation to sleep variables, with iRBD patients showing increased values compared to HCs in the following parameters: SOL N1, REM latency, REM density, and REM arousal index. We did not find significant differences between the two groups with respect to emotional assessment scales. Even though we did not find a difference in the overnight habituation between HCs and iRBD patients, nor an effect of the overnight habituation within each group, we did find interesting and significant relationships between overnight habituation and several sleep parameters. A negative significant correlation was found between overnight habituation and both WASO and REM latency, and a positive significant correlation with N2. In the HC group, overnight habituation positively correlated with REM duration. Regarding sleep microstructure, overnight habituation showed positive correlations with SS indices, a negative correlation with arousal and awakenings REM index, and a close to significant negative correlation with arousal index. A maladaptive relationship between emotion dysregulation and phasic events was also confirmed through questionnaires, revealing significant positive correlations between the DERS strategies subscale and REM phasic indices, including REM density and REM arousal index. Starting from the differences between the two groups, we did not replicate the results of previous studies showing significant differences between iRBD patients and HCs on emotional assessment scales [25,26]. While the small sample size may have contributed to this, it is important to note that we included iRBD patients with recent diagnoses and without any symptoms of overt neurodegeneration, which could affect the results. Concerning sleep variables, as expected, REM arousals and awakenings were higher in iRBD patients compared to HCs; indeed, according to the diagnosis, REM sleep in iRBD is disrupted by RSWA, leading to numerous phasic events. The negative correlation between overnight habituation and WASO is an interesting, albeit non-specific, finding. This result seems to indicate that non-continuous sleep, characterized by increased wakefulness, does not support proper emotional habituation processes. However, it does not offer specific relevant information about the roles of particular sleep stages and indices. The sleep stages that seem to play an important role in overnight emotional processes are N2 and REM sleep. Concerning N2, sleep spindles play a crucial role in HCs, but not in iRBD patients. Parietal SSs, particularly those in the fast frequency range, revealed greater correlations with overnight habituation. Accordingly, fast SSs are associated with a wide cortical activation encompassing areas involved in sensorimotor and memory consolidation processing [17]. Notably, significant alterations in SS activity have been reported in iRBD patients by previous studies [32,33] suggesting a possible breakdown of SS-related mechanisms in this disorder. Interestingly, SS alterations are considered one of the most consistent findings confirming a breakdown also of NREM sleep in these patients [34]. The important role of SSs in memory consolidation is renowned. These oscillations support the integration of newly acquired memories into existing knowledge schema through the reactivation of traces [16,35,36]. Importantly, a complementary role for the adequate processing of emotional memories seems to be played by REM sleep. Indeed, its duration supports the emotion habituation processes in HCs, and considering the whole sample, REM latency as well as REM phasic indices hinder the correct functioning. These results are in line with previous literature, confirming the important role of REM sleep in emotional habituation and its failure in the presence of restless REM sleep [1,10,16,37]. This study may provide an integrative perspective on the role of sleep in emotional processing, highlighting the crucial role of N2, particularly sleep spindles, as well as the REM stage. One of the key values of this paper lies in the inclusion of the iRBD group. iRBD, as a prodromal phase of synucleinopathies, offers a unique window for approaches aimed at slowing down phenoconversion. An early recognition of mood symptoms is important in the management of neurodegenerative disorders. The results of this paper may help to shed light on the relationship between sleep and the emotional brain in the context of ongoing neurodegenerative processes and it may pave the way for pharmacological approaches to stabilize REM sleep in iRBD patients, due to the overnight emotional habituation breakdown in presence of restless REM sleep.

Some limitations of this study need to be acknowledged. First, the small sample size limits the impact of our results. Second, the absence of autonomic measures, such as heart rate variability and skin conductance, could have provided objective indices for overnight habituation alongside subjective arousal responses. Moreover, the study relied on a single night to assess emotional habituation. Emotional habituation might require multiple nights for comprehensive observation and understanding [13]. In our single-night experiment, we did not observe a significant decrease in arousal ratings to negative images from pre-sleep to post-sleep, which may have not undermined the significant role of REM and N2 sleep but might reflect only an initial part of this complex process. Furthermore, it is worth noting a gender imbalance in the distribution between the two groups, with the iRBD group showing a higher male percentage compared to the HC group. Although this difference is not statistically significant, it should be considered that previous literature has reported greater self-reported ratings of negative stimuli in females compared to males. However, gender had no effect on overnight habituation. It might have resulted in lower ratings in the iRBD group. Nevertheless, it did not affect the night-to-morning difference but eventually only the absolute value of ratings. For this reason, it may have flattened some results rather than highlighting minor effects. Finally, the use of International Affective Picture System (IAPS) images, while largely employed for comparability in the literature, might be less ecologically valid for assessing arousal responses.

To conclude, it is important to note that our exploratory study raises numerous questions for future research. Future studies should consider larger sample sizes, utilize multiple nights of data collection, and incorporate the use of techniques such as targeted memory reactivation to manipulate the phenomenon of memory reactivation also in the context of iRBD. If future studies confirm our results, the beneficial role of SSs in NREM, and the detrimental role of REM phasic events on overnight habituation processes, this could pave the way for pharmacological approaches aimed at stabilizing REM sleep and increasing the density of sleep spindles.

## 4. Materials and Methods

### 4.1. Subjects

We enrolled 8 iRBD patients (7 male patients, mean age 68.25 ± 5.12 years) and 9 HCs (4 male subjects, mean age 60 ± 6.63 years). HCs and iRBD patients were recruited in two different centers (Cardiff University Brain Research Imaging Centre, Cardiff; Sleep Disorders Center, Vita-Salute San Raffaele University, Milan). Diagnosis of iRBD was performed by a neurologist expert in sleep medicine based on clinical information and PSG recording in accordance with the current clinical criteria defined by the International Classification of Sleep Disorder—third edition [24]. HC participants were recruited through online advertisements and screened for exclusion criteria using an online form. The exclusion/inclusion criteria for all participants, both HCs and iRBD patients, are listed below:

- No history of psychiatric, neurologic, or sleep disorders (other than iRBD in the pathological group) and no current use of psychoactive drugs or habitual use of marijuana. Not under the influence of any medication or substance that could directly affect sleep;
- Normal or corrected to normal vision and good sleep quality.

All subjects were adults and gave their written informed consent to the study. The study was approved by the Local Ethics Committee (protocol code EC.22.03.08.6546) and was conducted in accordance with the Declaration of Helsinki.

### 4.2. Procedure

**Figure 6:**
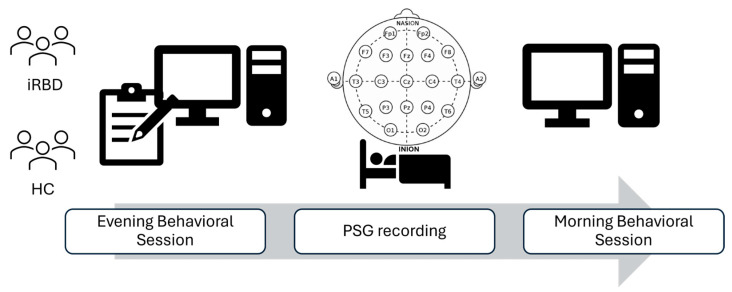
Experimental design: iRBD and HC groups completed two behavioral sessions and a PSG recording during the night in between. The evening session involved mood symptom scales and a computer-based arousal rating task; in the morning session, the task was administered again. HC = healthy controls; iRBD = isolated rapid eye movement behavior disorder; PSG = polysomnography.

The experimental design consisted of a single overnight session. In the evening, participants performed a questionnaire battery assessing mood functioning and an emotional arousal rating task (evening session part), followed by a night of sleep with PSG recording. In the morning, participants were asked to perform again the emotional arousal rating task (morning session part). Figure 6 illustrates the experimental design.

### 4.3. Materials

Questionnaires for the assessment of mood symptoms and emotional processing:

- Beck Depression Inventory (BDI) for the assessment of depressive symptoms [38]. The BDI is a self-report scale consisting of 21 items on a four-point scale assessing symptoms and attitudes. Higher scores reflect more severe depressive symptomatology;
- State-Trait Anxiety Inventory (STAI) for the assessment of anxiety [39]. The STAI includes two different scales: STAI-Y-1 measuring state anxiety, based on the subject feeling “at that moment”, and STAI-Y-2 measuring trait anxiety, based on the subject’s general feelings. Both scales include 20 items. Higher scores indicate higher levels of anxiety;
- Dimensional Apathy Scale (DAS) for the evaluation of apathy [40]. The DAS is a self-report questionnaire consisting of 24 items rated on a four-point Likert-type scale (from “hardly ever” to “almost always”). The scale assesses three apathy subtypes: (1) executive apathy, characterized by impairments associated with planning, attention, or organization; (2) emotional apathy, defined by difficulties in emotion integration; and (3) initiation apathy, related to the lack of motivation for self-generation of behaviors or cognition;
- DERS for the assessment of emotion dysregulation [41]. The DERS is a self-report inventory including six factors: (1) “non-acceptance of emotional responses”; (2) “difficulties in distracting with emotion and performing alternate behavior”; (3) “lack of confidence in emotional regulation skills”; (4) “difficulties in behavioral control”; (5) “difficulty in recognizing emotions”; and (6) “reduced emotional self-awareness”. Participants are asked to indicate their agreement with 36 items on a five-point Likert-type scale (from “almost never” to “almost always”). Higher scores indicate greater difficulties in regulating emotion.

### 4.4. PSG Recording

All PSG recordings were performed in a soundproof room. The electroencephalographic (EEG) signals were recorded from sintered Ag-AgCl electrodes according to the international 10–20 system (19 EEG channels: Fp1, Fp2, F3, F4, F7, F8, Fz, C3, C4, Cz, P3, P4, Pz, T3, T4, T5, T6, O1, and O2) with averaged mastoids reference. Electro-oculograms (EOGs) and submental electromyogram (EMG) were also recorded. Additional EMG electrodes in iRBD patients were positioned on the flexor digitorum superficialis (FDS) according to the indications provided by Sleep Innsbruck Barcelona (SINBAR) group for the evaluation of RSWA in iRBD patients [42] and on the anterior tibialis muscles for the identification of Periodic Limb Movements during sleep following the official World Association of Sleep Medicine criteria [43].

### 4.5. Sleep Stages

Sleep stages were scored visually in 30 s epochs according to standard criteria [44]. The following are considered as sleep macrostructure dependent variables: (a) total time spent in bed (TIB); (b) total sleep time (TST), defined as the sum of time spent in N1, N2, Slow Wave Sleep (N3), and REM; (c) a percentage of each sleep stage (time spent in a sleep stage/TST × 100); (d) SOL to N1 and N2; (e) WASO; (f) REM latency; and (g) sleep efficiency (SE = TST/TBT × 100).

#### 4.5.1. NREM Sleep Microstructure

SSs were automatically analyzed using YASA (v 0.6.1) [45]. SSs were identified as a burst of activity ranging from 12 to 15 Hz with a duration of more than 0.5 s. SSs were detected on central (i.e., Cz) and parietal (i.e., Pz) channels in N2. Then, we calculated SS density, defined as the total number of SSs occurring in N2 divided by the minutes spent in this sleep stage. Finally, we separated fast and slow spindles using the following frequency ranges: 12–13.5 Hz and 13.5–15 Hz [17].

#### 4.5.2. REM Sleep Microstructure

The eye movements were manually analyzed, following the guidelines in the AASM manual [44]. REMs were detected considering mini epochs of 3 s at a time. REMs were identified as conjugate and irregular eye movements, and having a peak with an initial deflection lasting less than 500 ms. REM density was computed by dividing the total number of REMs by the duration of REM sleep in minutes. We also detected arousals in REM sleep, which are defined as a sudden change in EEG frequency, including alpha, theta, and/or >16 Hz (except spindles), lasting for at least 3 s, with at least 10 s of stable sleep preceding the event, accompanied by concomitant increases in submental EMG amplitude lasting at least 1 s [44]. In addition to the arousal definition, the following guidelines were also considered: when the level of EMG in REM sleep appeared to fluctuate, the increase in EMG in the presumed arousal area had to exceed the background level of fluctuations. We applied the same rule to EEG and we determined the onset of arousal when a defined change in background EEG was observed. Furthermore, we assessed the number of awakenings occurring in REM sleep. REM arousal index was calculated as the number of arousals in REM sleep divided by the minutes spent in this sleep stage. REM arousals and awakenings index was calculated as the ratio of the total arousals and awakenings over the duration of REM sleep. To resume, the following were considered as REM sleep microstructure variables: (a) REM arousal index; (b) REM arousals and awakenings index; and (c) REMs density index (number of rapid eye movements in REM/time spent in REM). All the above indices were considered as phasic events occurring in REM sleep.

### 4.6. Experimental Task

Arousal rating task: Participants viewed 80 emotionally negative and neutral images, each presented once in a pseudorandom order (i.e., no more than two images with the same valence presented consecutively) to avoid potential emotional priming, expectation, or habituation effects. At the beginning of each trial, a central fixation cross was presented for 0.5 s. Then, the image was shown in the middle of the screen for 1 s. Afterward, the central fixation cross reappeared for a jittered interval, with a varied delay of 3.5 s, 4.5 s, 5.5 s, or 6.5 s (counterbalanced and randomized across trials). Simultaneously, a semantically related sound was presented through headphones for 1 s (400 ms × 2 repetitions separated by a gap of 200 ms), corresponding to the image on the screen. Participants rated each image-sound pair for emotional arousal on a continuous scale from one to nine (from “not arousing at all” to “extremely arousing/upsetting”). We selected images from IAPS [46] and sounds from either International Affective Digitized Sounds (IADS) [47] or from freely available internet sources (e.g., freesound.org, accessed on 1 February 2022). The arousal rating task was implemented in PsychoPy3 Builder (v2021.1.2). From the task data, we calculated the habituation of emotional reactivity to negative image-sound pairs from night to morning, which we called “overnight habituation”. This index was calculated as the difference between the evening mean arousal score and the morning mean arousal score of the negative image-sound pairs, divided by the evening mean arousal score for each subject.

### 4.7. Statistical Analyses

We conducted statistical analyses using IBM SPSS Statistics 20 and Jeffreys’s Amazing Statistics Program 0.18.3.0 (JASP, Amsterdam, NL, USA) software. Since the small sample size, we performed non-parametric tests. A *p*-value < 0.05 was considered statistically significant. In addition to the descriptive statistics, to assess the differences in overnight habituation, sleep architecture, REM and NREM sleep microstructure, and mood questionnaires between iRBD patients and the HC group, we utilized Mann–Whitney U tests. We applied the Mann–Whitney U test also to evaluate whether gender may have influenced overnight habituation. We performed a correlation analysis between sleep variables and age to assess whether age may have affected sleep parameters. Then, for significant correlations, we repeated the Mann–Whitney U test using age-corrected residuals. As regards REM indices, we tested the hypothesis that iRBD patients have a longer REM latency and a higher number of phasic events, according to their diagnosis. Instead, for all the remaining variables, we tested the hypothesis that the two groups differ. Additionally, we employed the Wilcoxon Signed-Rank Test to evaluate the differences between pre- and post-sleep arousal ratings to negative images separately for each group. Then, to explore the relationship between overnight habituation and REM sleep macrostructure (duration and latency), as well as between overnight habituation and REM sleep phasic indices (REMs density, arousals, and arousal and awakenings index), we performed Spearman’s correlations. Finally, Spearman’s correlations were also performed to assess the relationship between overnight habituation and sleep architecture variables, as well as between overnight habituation and sleep spindles. As regards REM indices, we tested the hypothesis that REM duration supports overnight habituation while phasic events disrupting this stage as well as an increase in REM latency hinder overnight habituation. Concerning the relationship between overnight habituation and all the remaining variables, we tested whether they correlated.

## Figures and Tables

**Table 1:** PSG sleep parameters for the HC and patients’ groups.

PSG Parameters	Group	Mean ± SD	Mean Rank	U Statistic	*p*
SE	HC	83.47 ± 13.26	9.25	26	0.574
	iRBD	77.13 ± 18.27	7.75		
TIB	HC	497.50 ± 41.78	10	20	0.234
	iRBD	469.69 ± 19.25	7		
TST	HC	417.56 ± 84.76	10.50	16	0.105
	iRBD	358.50 ± 86.79	6.50		
WASO	HC	68.06 ± 51.39	7.38	33	0.613
	iRBD	98.29 ± 83.38	8.71		
SOL N1	HC	4.44 ± 4.13	5.44	48.50	0.014 *
	iRBD	10.79 ± 3.59	10.93		
SOL N2	HC	16.06 ± 10.83	5.88	45	0.054
	iRBD	41.14 ± 29.89	10.43		
%N1	HC	17.08 ± 8.65	7.88	37	0.645
	iRBD	21.77 ± 10.70	9.12		
%N2	HC	57.88 ± 8.99	10.25	18	0.161
	iRBD	50.33 ± 9.80	6.75		
%N3	HC	7.21 ± 10.26	9.25	26	0.574
	iRBD	5.21 ± 8.67	7.75		
%REM	HC	17.82 ± 4.00	6.88	45	0.195
	iRBD	22.69 ± 8.33	10.12		
REM latency	HC	99.50 ± 47.01	6.38	49	0.041 *
	iRBD	182.81 ± 102.13	10.62		
REMs density	HC	3.22 ± 1.46	4.50	56	<0.001 *
	iRBD	8.19 ± 2.82	12		
REM arousal index	HC	0.23 ± 0.10	6	44	0.036 *
	iRBD	0.35 ± 0.11	10.29		
REM arousal and awakening index	HC	0.29 ± 0.12	6.75	38	0.281
	iRBD	0.38 ± 0.12	9.43		
SS density N2 Cz	HC	1.10 ± 1.12	9	20	0.397
	iRBD	0.59 ± 0.64	6.86		
SS density N2 Pz	HC	1.65 ± 1.60	8.62	23	0.613
	iRBD	1.22 ± 0.98	7.29		

HC: healthy control; iRBD: isolated rapid eye movement behavior disorder; N1: sleep stage 1; N2: sleep stage 2; N3: sleep stage 3; PSG: polysomnography; REM: rapid eye movements; SD: standard deviation; SE: sleep efficiency; SOL: sleep onset latency; SS: sleep spindle; TIB: time in bed; TST: total sleep time; WASO: wake-after-sleep onset. * *p*-value < 0.05.

**Table 2:** Mood questionnaires scores in HC and iRBD groups.

PSG Parameters	Group	Mean ± SD	Mean Rank	U Statistic	*p*
DERS Total	HC	70.37 ± 17.83	7.75	38	0.574
	iRBD	76.75 ± 17.55	9.25		
DERS Non-Acceptance	HC	10.00 ± 3.25	6.69	46.50	0.130
	iRBD	13.00 ± 3.89	10.31		
DERS Goals	HC	13.87 ± 5.38	9.56	23.50	0.382
	iRBD	11.50 ± 2.73	7.44		
DERS Impulsiveness	HC	9.50 ± 4.21	6.75	46	0.161
	iRBD	13.37 ± 5.10	10.25		
DERS Awareness	HC	14.62 ± 5.34	9.50	24	0.442
	iRBD	12.12 ± 5.36	7.50		
DERS Strategies	HC	14.37 ± 4.69	6.69	46.50	0.130
	iRBD	17.75 ± 4.68	10.31		
DERS Clarity	HC	8.00 ± 2.51	8.44	32.50	1.00
	iRBD	8.87 ± 4.61	8.56		
BDI	HC	5.37 ± 5.34	7.75	38	0.574
	iRBD	6.00 ± 2.39	9.25		
STAI-Y1	HC	29.37 ± 8.42	7.50	40	0.442
	iRBD	34.57 ± 13.38	9.50		
STAI-Y2	HC	32.37 ± 12.12	7.38	41	0.382
	iRBD	34.25 ± 7.46	9.62		
DAS Executive	HC	7.62 ± 4.81	10.81	13.50	0.057
	iRBD	3.62 ± 1.92	6.19		
DAS Emotional	HC	7.37 ± 4.63	8.25	34	0.878
	iRBD	7.37 ± 3.20	8.75		
DAS Behavior and Cognition	HC	9.25 ± 3.99	9.38	25	0.505
	iRBD	6.62 ± 4.14	7.62		
DAS Total	HC	24.25 ± 7.05	10.50	16	0.105
	iRBD	17.62 ± 7.11	6.50		

BDI: Beck depression inventory; DAS: dimensional apathy scale; DERS: difficulties in emotion regulation scale; HC: healthy controls; iRBD: isolated rapid eye behavior disorder; PSG: polysomnography; SD: standard deviation; STAI: State-Trait Anxiety Inventory.

## Data Availability

Data are available from C Leitner (caterina.leitner@hotmail.com/c.leitner@studenti.unisr.it) upon request.
